# Mapping human augmentation technologies for societal impact: A multilevel framework for classification and innovation potential

**DOI:** 10.1371/journal.pone.0343292

**Published:** 2026-02-25

**Authors:** Yousif Elsamani, Cristian Mejia, Yuya Kajikawa

**Affiliations:** 1 Institute for Future Initiatives, The University of Tokyo, Tokyo, Japan; 2 Department of Innovation Science, School of Environment & Society, Institute of Science Tokyo, Tokyo, Japan; King Fahd University of Petroleum & Minerals, SAUDI ARABIA

## Abstract

While human augmentation (HA) technologies offer significant potential for addressing societal challenges, their specific application towards social goals like sustainability, quality of life, and well-being remains understudied. This study addresses this gap by investigating the central research question: How do current HA research landscapes align with global social targets, and what are the comparative advantages of these technologies across individual, organizational and societal levels? To answer this, we employ a comprehensive bibliometric analysis of 6,284 papers across 60 specific research topics. Using citation network analysis and text similarity mapping, we reveal that although HA literature is smaller in volume, it exhibits high topical diversity and distinct specialization. Semantic mapping identifies selective but significant intersections between HA research and social domains, particularly through three influential subclusters: Industrial Worker Augmentation Systems, Human-Building Interface Assessment Technologies, and Assistive Augmentation Technologies. Based on these findings, we developed a novel nine-dimensional performance framework for multi-level evaluation of HA’s societal value. The practical applicability of this framework is validated through real-world case studies, including industrial exoskeleton deployments at Ford and VR training at Walmart, demonstrating tangible gains in productivity, safety, and learning acceleration. This framework offers actionable insights for assessing HA’s social impact, guiding responsible innovation, and identifying commercialization opportunities aligned with well-being, productivity, and sustainability.

## 1. Introduction

Human augmentation (HA) technologies have emerged as a transformative field at the intersection of science, technology, and societal needs. Leveraging advanced systems such as augmented reality (AR), artificial intelligence (AI), Internet of Things (IoT), and wearable devices, HA technologies aim to enhance physical, cognitive, and sensory capabilities [1–3]. The applications of HA span diverse domains, including healthcare, education, sports training, rehabilitation, daily life support, and enhancement of skill acquisition [4,5], addressing critical societal challenges such as aging, disability, and workforce productivity. Unlike robotics, which often operates autonomously, HA integrates seamlessly with human control systems, effectively acting as an extension of the human body [6]. This distinction underscores the unique role of HA in enabling individuals to overcome limitations and enhance overall performance.

HA technologies can enhance cognitive, sensory, and motor functions but also pose risks like information overload, increased cognitive load, and reduced motor efficiency due to excessive demands or inadequate device design [7–9]. The field has seen a proliferation of studies focusing on technical advancements and specific applications; however, a comprehensive understanding of its societal impacts and its comparative advantages is still lacking.

While existing literature on HA offers insights into its technical and application aspects, key gaps remain. A comprehensive overview linking HA to societal issues, such as sustainability, well-being, and quality of life, is lacking. Research has largely focused on related or sub-fields like human-computer interaction and virtual reality but has overlooked HA’s social dimensions, including its potential to drive behavioral change, enrich leisure activities, and promote social sustainability. Moreover, there is no established evaluative framework to assess the comparative advantages of HA technologies across critical performance dimensions. Developing such a framework could provide a structured approach to advance the field and guide future innovation. Given the study’s emphasis on societal outcomes, academic literature offers a richer foundation than other data (e.g., patent data), as it typically provides deeper conceptual insights, interdisciplinary perspectives, and evidence-based evaluations that are often absent in commercially oriented patent filings.

In framing the societal relevance of human augmentation, it is essential to situate its potential impacts within global policy contexts. The selection of sustainability, well-being, and quality of life as the study’s focal dimensions is grounded in their central role within global development agendas, particularly the United Nations Sustainable Development Goals (SDGs). Goals such as SDG 3 (Good Health and Well-being), SDG 8 (Decent Work and Economic Growth), SDG 9 (Industry, Innovation and Infrastructure), SDG 10 (Reduced Inequalities), and SDG 12 (Responsible Consumption and Production) collectively underscore the imperative to balance technological advancement with human and environmental flourishing [10,11]. Human augmentation technologies, by extending physical and cognitive capacities, have the potential to contribute directly to these objectives through improved health outcomes, inclusive participation in innovation-driven economies, and more sustainable modes of living and working. However, despite these broad alignments, systematic frameworks that evaluate how HA contributes to such sustainability and well-being goals remain underdeveloped. Addressing this gap is essential to understand the comparative societal value of HA technologies and to guide their responsible integration into future innovation systems.

This article seeks to address these gaps by providing a comprehensive analysis of HA research, with a focus on its applications and societal impacts. Specifically, it aims to review the current state of HA research, embodied in academic literature, and its applications in addressing social issues related to well-being, sustainability, and quality of life. Furthermore, it seeks to develop a framework for assessing the comparative advantages of HA technologies using key performance dimensions (KPD). Finally, the study aims to highlight the potential commercialization opportunities for HA technologies by using emerging technology signals to be identified through cluster analysis and their cross-disciplinary links to social impact areas.

This study advances the field of Human Augmentation through three distinct contributions. First, it provides a comprehensive analysis explicitly linking HA technologies with societal outcomes related to well-being, quality of life, and sustainability. This moves beyond the purely technical focus of prior bibliometric studies to address critical gaps in existing research. Second, it addresses the lack of structured assessment tools by developing a novel nine-dimensional Key Performance Dimension (KPD) framework, grounded in empirical data, to assess the comparative advantages of HA technologies. Third, by systematically mapping technological advancements to social impact metrics, the research identifies promising but underexplored applications, guiding future innovation toward meaningful societal benefits.

The remainder of this paper is organized as follows. Section 2 reviews the existing literature on HA categorizations and prior bibliometric analyses. Section 3 details the data collection from Web of Science and the methodological approach using citation network analysis and semantic mapping. Section 4 presents the results of the clustering and semantic linkage analysis. Section 5 discusses the development of the comparative advantages framework and validates it against real-world applications. Finally, Section 6 concludes the study with limitations and recommendations for future research.

## 2. Literature review

While HA technologies broadly encompass augmentation of human abilities [1], distinct categorizations further clarify their intended applications. De Boeck and Vaes [12] categorize these into restoring, supplementing, or exceeding human capabilities. This typology has practical implications across fields such as education [13], sports training [14], rehabilitation [15], skill acquisition [16], and daily activity support [17]. Expanding on these categories within industrial contexts, Romero et al. introduced the “Operator 4.0” typology, which classifies augmentation into physical (e.g., exoskeletons), sensory (e.g., smart sensors), and cognitive (e.g., augmented reality) dimensions designed to execute “smart work” rather than manual labor [18]. This industrial perspective is crucial as it reframes HA not merely as a medical intervention but as a driver of workforce adaptability.

Prattichizzo et al. notably explore supernumerary robotic limbs (SRLs), highlighting their direct integration within human control systems rather than autonomous operation [19]. Leigh et al. extend this, proposing human-robot symbiosis as distinct from standalone robotics, advocating collaborative augmentation paradigms [20]. Collectively, these classifications have advanced our understanding of HA’s operational and design dimensions but remain largely technology-centric. Few attempts have examined how these categories translate into measurable social or behavioral outcomes. This limitation highlights the need for a more integrative framework capable of linking technical functions with societal value.

### 2.1. Past bibliometric analyses on HA

Bibliometric analysis has proven instrumental in mapping the research landscape and identifying trends. Many studies discussed these methods and provided a comprehensive overview of bibliometric methodologies, highlighting their role in understanding the evolution of research domains and emerging research areas [21,22]. Chang et al. conducted bibliometric analysis of human enhancement technologies over the past two decades reveals evolving research themes from disease treatment to general augmentation, a shift from cognitive to ethical and emotional enhancements, and a progression from drug-based to biotechnological approaches, highlighting an interdisciplinary convergence across medicine, neuroscience, psychology, and ethics [23]. Other domain-specific reviews have focused on the safety and ergonomic implications of collaborative technologies, noting a sharp rise in research regarding physical risk reduction in human-robot collaboration [24].

Similar studies focused on reviewing related topics such as human-computer interaction were key topics such as virtual reality, augmented reality and video games are identified [25]. Another example of bibliometric studies on related topics can be found in the field of human-building interaction. Recent research shows a steady increase in publications, particularly since 2016, and emphasizes that future work should focus on the entire building life cycle, enhancing structural performance, and improving decision-making during the construction process [26].

While prior bibliometric studies have successfully mapped thematic trends and disciplinary linkages, they often remain descriptive, focusing on the evolution of research topics rather than their social implications. None of these studies systematically connect technological trajectories with human-centric outcomes such as well-being or sustainability. This methodological gap motivates the need for a comparative framework that bridges bibliometric mapping with evaluative dimensions of societal impact.

### 2.2. Societal outcome dimensions in HA

The selection of well-being, quality of life, and sustainability as the focal societal outcomes in this study is grounded in both global development priorities and a growing body of interdisciplinary research. These domains are central to evaluating the societal value of emerging technologies and align closely with international policy frameworks such as the United Nations Sustainable Development Goals (SDGs) [27]. By situating HA research within these pillars, we emphasize its potential to drive responsible and inclusive innovation.

Well-being serves as a foundational lens for understanding the human-centric promise of HA technologies. Many HA innovations are explicitly designed to support individuals’ physical, cognitive, and emotional health. Devices such as neuroprosthetics, rehabilitation systems, and wearable biosensors have been shown to reduce cognitive load, enhance recovery, and enable independent living, particularly among aging populations and persons with disabilities [28,29]. In this regard, HA technologies move beyond performance enhancement toward supporting holistic human flourishing, empowerment, and resilience in everyday life. This shift aligns with the emerging paradigm of Industry 5.0, which prioritizes human-centricity and resilience over pure efficiency, positing that technology should adapt to human needs rather than requiring workers to adapt to machines [30].

Building upon well-being, quality of life represents a broader goal encompassing individual autonomy, social participation, and access to meaningful activities. HA tools such as assistive communication systems, augmented reality applications, and skill-enhancing interfaces have demonstrated their ability to reduce functional limitations and foster inclusion [28,29,31]. Improvements in mobility, interaction, and engagement not only enhance individual capabilities but also create ripple effects in social and community settings. Prioritizing the quality of life ensures that HA technologies are not only innovative but also empathetic and designed with attention to real-world human needs and lived experiences.

Finally, sustainability offers a critical systems-level perspective for evaluating the broader impacts of HA technologies. Sustainability, particularly its social and environmental dimensions, frames innovation in terms of long-term viability and ethical responsibility [32]. Recent studies highlight how digital and smart technologies, including Internet of Things (IoT) systems, can be leveraged to advance sustainability in healthcare, mobility, and the built environment [31]. HA’s tight integration with human behavior and context positions it well to contribute to sustainable practices through energy-aware design, behavioral nudging, and context-sensitive workforce support tools. These applications underscore HA’s capacity to support societal transitions toward more sustainable modes of living and working. By anchoring this study on well-being, quality of life, and sustainability, we propose an evaluative framework that connects technological advancement with measurable human and environmental benefits. This alignment not only enhances the relevance of HA research but also ensures its outcomes resonate with the pressing challenges and aspirations of contemporary society.

Existing studies acknowledge HA’s potential to improve health, autonomy, and inclusion, yet they tend to treat well-being, quality of life, and sustainability as secondary effects rather than core evaluative criteria. This study repositions these dimensions at the center of analysis, not as by-products but as primary indicators for assessing the desirability and legitimacy of HA innovations.

### 2.3. Technology and social issue mapping studies

Boeck et al. categorized HA into sensory, physical, cognitive, and social augmentation across three dimensions: replicating, supplementing, and exceeding human abilities [28]. Such categorization can enable product designers to better understand and characterize the type of HA products. Notably, social augmentation remains underexplored compared to other categories, necessitating greater focus in future research [28]. In line with this concern, recent studies have elucidated the complex relationship between technological advancements and societal challenges. For instance, a literature-based discovery (LBD) approach has demonstrated how technologies, such as robotics, contribute to addressing critical social issues like healthcare and elderly well-being, particularly in surgery, rehabilitation, and companionship [33]. The LBD method identifies plausible linkages between technological domains and social needs and offers insights for decision-makers to prioritize research targets and inform innovation policies [33]. Such methodologies underscore the importance of systematic approaches to uncovering the societal impacts of emerging technologies. Similarly, topic modeling of news articles combined with academic citation analysis has been used to map robotic technologies to country-specific social challenges [34]. By linking social issues, such as Japan’s aging population and nuclear energy concerns, to advancements in companion robotics and robots for hazardous environments, this approach highlights the potential for real-time societal needs assessment through data mining and content analysis [34]. This methodology not only aids policymakers in identifying suitable technological solutions but also enables researchers to explore innovation frontiers based on emerging societal demands. Moreover, El Khatib et al. emphasize how digital modernization and IoT technologies advance social sustainability, enhance quality of life, and align with global sustainability goals [31]. Likewise, Manero et al. highlight the ethical dimensions of technology implementation, advocating for sustainable designs in neuroprosthetics and bionic technologies to prevent obsolescence and ensure long-term usability [29]. These emerging methodologies collectively demonstrate how data-driven approaches can illuminate the link between technology and social needs. However, they typically analyze discrete technologies or domains rather than constructing a unified comparative framework applicable across multiple HA modalities and societal outcomes.

Despite these attempts, a significant gap remains in exploring the intersection between advanced technologies, such as HA, and pressing social issues, including sustainability, well-being, and quality of life. Addressing this gap is crucial for uncovering the transformative potential of HA technologies and their broader societal implications. As demonstrated by the LBD approach [33] and topic modeling techniques [34], integrating diverse analytical methodologies can illuminate how emerging technologies can be aligned with societal needs, driving meaningful innovation and policymaking. While prior research has explored HA through technical advancements and specific applications, our study offers a systematic, large-scale analysis that directly links HA technologies to societal issues. Additionally, we introduce a novel framework to evaluate the comparative advantages of HA technologies across KPDs. This dual focus on semantic alignment and impact prioritization distinguishes our work, offering actionable insights for guiding research, policy, and commercialization toward HA applications that address pressing human needs.

## 3. Data and methods

### 3.1. Data

We utilized the Web of Science (WoS) database for data collection, given its comprehensive coverage of high-quality academic publications across disciplines, rigorous indexing standards, and extensive metadata that facilitates citation network analysis [22]. WoS was selected over alternative databases for several key advantages: (1) superior citation linking capabilities essential for our network analysis methodology, (2) consistent metadata quality across disciplines enabling reliable DOI and bibliographic matching, (3) comprehensive coverage of interdisciplinary research spanning engineering, social sciences, and sustainability studies, and (4) standardized author keyword indexing crucial for our semantic analysis [35,36]. We followed a structured approach tailored to each research domain. For the HA dataset, we performed a topic search that collected all available data in the database, including citing articles that referenced publications matching our query terms. Given the large nature of the social issues under investigation, we implemented constraints for sustainability, wellbeing, and quality of life datasets. Specifically, to ensure currency, we restricted these searches to articles published within the past five years. We also searched for explicit mention of the query terms in the article title. This ensures that selected articles explicitly addressed these social issues as primary concerns rather than tangential considerations. Across all datasets, we limited our collection to academic article types. The specific query terms used for each domain and the resulting dataset sizes are presented in Table 1. Data was sourced on December 12, 2024. A detailed breakdown of dataset construction, including details of the query syntax and initial query hits, exclusions during processing, and final node counts for each network, is provided in Supplementary Materials (S1 Table in S1 File). Web of Science category filters, and export settings are documented in Supplementary Materials (S2 and S3 Tables in S1 File) to ensure full reproducibility.

**Table 1:** Query terms per dataset.

Dataset	Query	Records
HA	TS= (“human* augmentation” OR “human* enhancement” OR “augmenting human*” OR “enhancing human*”)	10,957
Sustainability	TI = “sustainability”	28,128
Wellbeing	TI = (“wellbeing” OR “well-being” OR “well being”)	26,281
Quality of Life	TI = “quality of life”	43,775

Our query strategy prioritized precision over recall by targeting researchers who explicitly acknowledge their work within these specific domains. For HA, we used broad synonymous terms title/abstract searches to capture the diverse terminology used in this emerging field. For the three social domains, the title-only search strategy prioritizes precision over recall by identifying studies where these concepts represent the primary research focus rather than peripheral considerations. This approach ensures our semantic linkage analysis captures researchers who explicitly position their work within these domains, enabling more meaningful comparison with HA literature. This approach ensures our analysis captures researchers who consciously position their work within these domains, enabling more meaningful semantic linkage analysis between fields where authors explicitly acknowledge working on these societal challenges.

### 3.2. Methods

For each of the four datasets, we constructed direct citation networks where academic articles functioned as nodes, with connections established when one article cited or was cited by another article in the dataset. This approach has been validated in previous bibliometric studies as an effective method for extracting robust taxonomies of research fields and identifying academic fronts [37].

To establish these connections, we utilized the reference lists provided as part of the metadata of the extracted records, which are found in the “Cited References” (CR) column in WoS. We matched references to articles in each dataset primarily through the document object identifier (DOI). In cases where articles lacked a DOI, matching was performed by comparing author names, publication year, journal title, volume, and issue number. This direct citation approach was selected over alternative citation-based networks (e.g., bibliographic coupling or co-citations) due to its demonstrated effectiveness in extracting meaningful taxonomies of research fields [38]. After constructing the networks, we extracted only the giant component from each network to ensure that all analyzed articles were connected to the main research corpus. Articles that matched our search queries but remained unconnected to the giant component were excluded from further analysis as they likely represented tangential research.

To identify distinct research topics within each domain, we applied the Louvain community detection algorithm [39] implemented in the Python-igraph library (version 0.11.3) with resolution parameter γ = 1.0 to divide each network into clusters. This algorithm was selected for its ability to effectively handle large networks and produce interpretable clustering solutions that maximize modularity, ensuring more connections exist within clusters than between them [40]. The Louvain method is particularly advantageous compared to other modularity maximization algorithms (e.g., Newman’s algorithm) as it generates fewer, more substantial clusters rather than a mix of very large clusters followed by numerous small ones, facilitating a more coherent interpretation of network trends. To achieve topic specificity, we repeated the clustering process over each cluster to obtain subclusters. Specifically, we applied two levels of Louvain clustering: the first pass identified major thematic domains (clusters), and the second pass was applied independently to each major cluster to identify specialized research areas (subclusters). The choice of resolution parameter and algorithm was validated through systematic comparison with alternative methods, as documented in Supplementary Materials (S4 and S5 Tables in S1 File). This allows us to have a hierarchy, where the main cluster represents a larger domain within each dataset, and the subcluster narrower research areas.

For each resulting cluster and subcluster, we calculated the number of articles, average publication year, and average citation count. We then labeled clusters based on their most common keywords and the content of their most-cited articles. To examine the relationship between HA research and the three social domains, we conducted a semantic linkage analysis by comparing vocabulary across clusters from different networks. This approach allowed us to identify topical overlaps and potential synergies between HA technologies and each social domain. To do so, we employed a sentence-transformer approach based on BERT (Bidirectional Encoder Representations from Transformers) to convert these aggregated cluster texts into dense vector representations [41]. Unlike traditional bag-of-words approaches that treat words as independent units, BERT-based transformers process text bidirectionally, allowing the model to understand words in context by considering both preceding and following terms. This contextual understanding enables the model to capture polysemy (words with multiple meanings), semantic relationships, and domain-specific terminology that might be missed by simpler vectorization methods.

**Fig 1:**
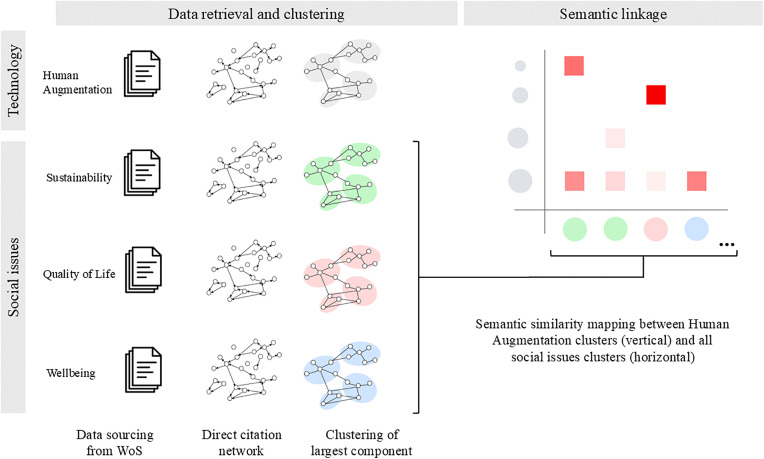
Methodological framework for analyzing semantic linkages between HA technologies and social domains.

We implemented the semantic similarity analysis using the sentence-transformers library [42] with the ‘all-MiniLM-L6-v2’ model, which generates 384-dimensional dense vector representations. Text preprocessing included standard tokenization and normalization procedures. For each cluster, we aggregated the abstracts and titles of all constituent articles into a single text representation, creating a comprehensive thematic profile of each research domain. Cosine similarity scores between HA subclusters and social domain subclusters were calculated using the resulting vector representations, with scores ranging from 0 (no similarity) to 1 (perfect similarity). The resulting similarity scores were visualized as a heatmap (Fig 1), where the y-axis represented HA clusters and the x-axis displayed social issue clusters, with the intersections indicating cosine similarity. This visualization allowed us to identify potential areas of convergence between HA technologies and various social challenges, as well as gaps that might represent opportunities for future research integration. Model selection was validated by comparing semantic similarity matrices across multiple embedding architectures, which demonstrated high inter-model correlation (r > 0.83), confirming robustness of the semantic linkage patterns to model choice (Supplementary Materials, S5 Table in S1 File). To identify semantically meaningful linkages, we defined ‘strong’ connections as those with a similarity score >= 0.3609, being the set of pairs identified as outliers in the similarity distributions given the comparatively high values across all possible HA-social domain cluster pairs. This threshold represents the top cross-domain semantic relationships, ensuring that highlighted connections reflect substantive thematic overlap rather than weak or spurious associations. The complete distribution of similarity scores is presented in Supplementary Materials (S1 Fig in S1 File).

## 4. Results

We collected four datasets of academic literature related to HA, sustainability, wellbeing, and quality of life. These datasets were analyzed using a citation network approach that enabled the identification of distinct research clusters and subclusters within each domain. Table 2 presents the characteristics of each network, including the number of nodes (articles), major clusters, and subclusters.

**Table 2:** Clustering results.

Dataset	Nodes	Clusters	Subclusters
HA	6,284	30	60
Sustainability	28,128	18	257
Wellbeing	26,281	19	186
Quality of Life	43,775	37	296

The analysis revealed substantial differences in the size and structure of the four research domains. The HA literature, with 6,284 articles, represents the smallest corpus, yet exhibits a relatively high degree of topical diversity with 30 major clusters subdivided into 60 subclusters. This suggests that HA research, while more limited in volume, encompasses a diverse range of specialized research directions. In contrast, the three social domains demonstrate significantly larger literature bases, with quality of life being the most extensive at 43,775 articles organized into 37 major clusters and 296 subclusters. This volume reflects the multifaceted nature and widespread academic interest in quality of life research across disciplines. Similarly, both sustainability (28,128 articles) and wellbeing (26,281 articles) represent robust research domains with comparable sizes but different structural characteristics: sustainability exhibiting fewer major clusters (18) but more granular subdivision into subclusters (257), while wellbeing presents slightly more major clusters (19) with fewer subclusters (186). These variations in network structure suggest different patterns of knowledge organization and specialization across the four domains. Additional statistics covering average publication years, average citations, and the names of clusters and subclusters are provided as supplementary materials.

**Fig 2:**
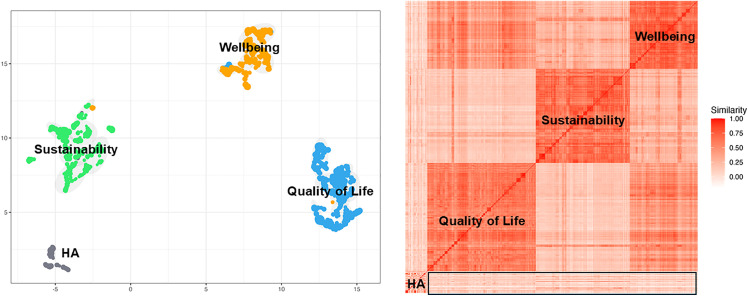
Topic model visualization (left) and semantic linkage (right) analysis of HA and social domains.

Fig 2 presents a visual representation of our analysis. The left panel displays a topic model visualization where the four datasets are plotted at the subcluster level using dimensionality reduction techniques. This visualization reveals the distinct positioning of the four research domains in the semantic space, with minimal overlap between them. HA (gray) appears as a relatively compact group in the lower left quadrant, indicating a more cohesive and specialized research focus. In contrast, the three social domains occupy different regions of the semantic space: sustainability (green) in the upper left quadrant, wellbeing (orange) in the upper right quadrant, and quality of life (blue) in the lower right quadrant. This spatial separation suggests fundamentally different research vocabularies, methodologies, and conceptual frameworks across the four domains, with limited natural integration in the current literature.

The right panel presents a heatmap of semantic linkages between all subclusters across the four datasets, with the black-bordered rectangle highlighting the specific intersections between HA and the three social domains. This focused section of the heatmap reveals several important patterns. First, the overall semantic similarity within the black-bordered rectangle shows predominantly lighter shades with scattered areas of more intense red, indicating that while there are some meaningful semantic connections between HA and social domains, these connections are selective rather than pervasive. The most pronounced semantic linkages appear between certain HA subclusters and sustainability topics, as evidenced by the more intense red regions of the highlighted rectangle.

**Fig 3:**
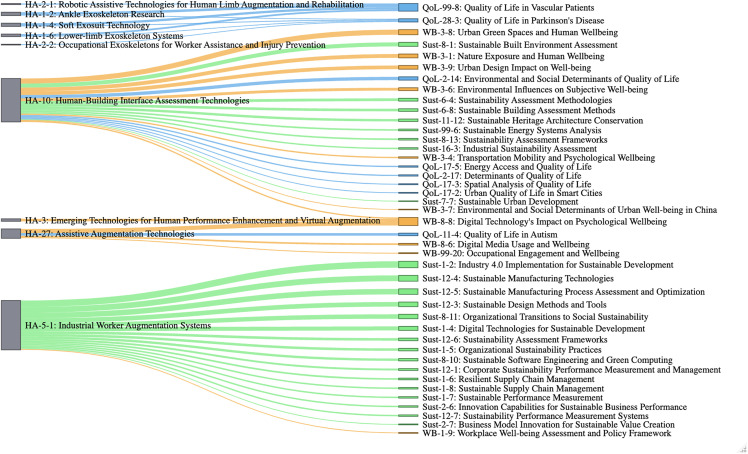
Top 50 strongest semantic connections between HA and social domain subclusters.

Fig 3 presents a Sankey diagram visualizing the strongest semantic connections between HA subclusters and social issue domains. This visualization confirms and elaborates on the patterns observed in the heatmap, providing a more detailed view of the specific research topics that have the highest potential for integration. We can observe different patterns of the linkages between those. Some HA technologies jointly contribute to a specific issue, and others have broader connections with social issue clusters.

The diagram reveals several key insights. First, three HA subclusters emerge as particularly influential topics with numerous connections to social domains: “Industrial Worker Augmentation Systems” (HA-5–1), “Human-Building Interface Assessment Technologies” (HA-10), and “Assistive Augmentation Technologies” (HA-27). Of these, “Industrial Worker Augmentation Systems” demonstrates the strongest and most numerous connections, primarily to sustainability subclusters, with similarity scores reaching as high as 0.598 with “Industry 4.0 Implementation for Sustainable Development” (Sust-1–2). This suggests that the integration of HA into industrial settings represents a particularly well-developed research area with strong conceptual alignment to sustainability objectives.

Second, the Sankey diagram confirms the predominance of sustainability connections, which constitute the majority of the top 50 semantic linkages. The result is consistent with that of Fig 2. Specifically, “Industrial Worker Augmentation Systems” shows strong semantic similarity with multiple sustainability subclusters focused on manufacturing, industry 4.0, and organizational sustainability. These connections highlight how HA technologies in industrial contexts are already conceptually aligned with sustainability goals in manufacturing processes, supply chain management, and corporate performance metrics, while some of these are related with sustainability of business and sustainable competitive advantage rather than environmental and social sustainability.

Third, the “Human-Building Interface Assessment Technologies” subcluster demonstrates remarkable versatility by forming significant connections across all three social domains. This subcluster shows particularly strong semantic similarity with wellbeing topics related to urban design and environmental influences on wellbeing (e.g., “Urban Green Spaces and Human Wellbeing” with a similarity score of 0.557) but also connects to sustainability assessment methodologies and quality of life determinants. This suggests that technologies designed to enhance human-building interactions represent a promising bridge between HA and multiple social domains.

Fourth, exoskeleton and assistive technologies (HA-1–2, HA-1–4, HA-1–6, HA-2–1, HA-2–2) demonstrate specific connections to quality of life subclusters related to medical conditions, particularly “Quality of Life in Vascular Patients” and “Quality of Life in Parkinson’s Disease.” These connections highlight the medical applications of HA technologies and their potential to address specific health-related quality of life challenges, while there is a possibility that the HA application is not limited to these two diseases.

Finally, the diagram reveals that technologies focused on “Assistive Augmentation” and “Emerging Technologies for Human Performance Enhancement” show notable connections to wellbeing subclusters related to digital technology impacts, suggesting an emerging research focus on how digital augmentation technologies influence psychological wellbeing and mental health outcomes.

**Fig 4:**
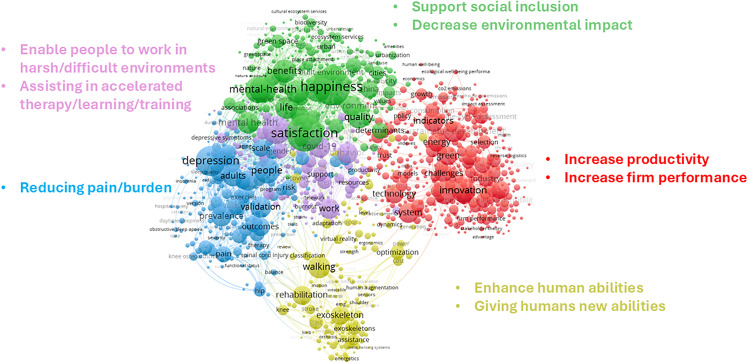
Keyword Co-Occurrence Network of HA Research Aligned with Sustainability, Wellbeing, and Quality of Life.

In addition to the analysis of semantic linkages between research domains, Fig 4 presents a keyword co-occurrence network derived from the highly similar intersecting clusters between HA and the social issue domains. This visualization provides a complementary perspective to our previous analyses by revealing the conceptual landscape where HA research overlaps with sustainability, wellbeing, and quality of life concerns.

In this network, keywords are connected when they co-occur within the same document, with connection strength proportional to co-occurrence frequency. The network was clustered using the same Louvain community detection algorithm employed in our citation network analysis, resulting in distinct thematic communities that represent key categories where HA intersects with social domains. This analysis allows us to go beyond identifying mere structural connections between research clusters to understand the specific conceptual dimensions where HA technologies engage with social challenges. While the previous analyses identified which research subclusters demonstrate semantic similarity, this network reveals the actual concepts, applications, and performance dimensions that constitute these connections.

Visualization in Fig 4 reveals five categories, distinguished by color and spatial positioning. Each category represents a distinct functional orientation or performance dimension of HA technologies in relation to social domains: The red cluster (right side) focuses on productivity enhancement and organizational performance, indicating that HA technologies are substantially conceptualized in terms of their capacity to increase productivity and improve firm performance. The yellow cluster (bottom) centers on human capability enhancement, with keywords suggesting applications that both enhance existing human abilities and provide entirely new capabilities. The blue cluster (bottom left) emphasizes pain reduction and burden alleviation, reflecting the medical and therapeutic applications of HA technologies. The pink cluster (upper left) highlights applications enabling work in harsh or difficult environments, suggesting protective and assistive functions of augmentation technologies. This section also covers accelerated therapy, learning, and training applications, revealing the educational and rehabilitative potential of HA technologies. The green cluster (upper right) captures social inclusion and environmental impact dimensions, indicating how augmentation technologies intersect with broader social and ecological concerns.

## 5. Discussion

The bibliometric analysis reveals a striking structural dichotomy: while HA research is technically robust, it remains semantically distinct from the core social domains of sustainability, well-being, and quality of life (as evidenced by the spatial separation in Fig 2). This separation suggests that HA innovation has historically followed a “capability-first” trajectory—focusing on technical feasibility—rather than a “society-first” approach. However, the strong semantic bridges identified in the “Industrial Worker Augmentation” cluster indicate a shifting paradigm. The prominence of this cluster validates the transition toward the “Operator 4.0” concept, where technology is increasingly viewed not just as a production tool, but as a means to ensure worker safety and inclusion in smart environments [18]. Furthermore, the strong sustainability linkages within this industrial cluster (Fig 3) suggest that the field is moving toward the principles of Industry 5.0, where human-centricity and resilience become drivers of technological adoption [30]. These bibliometric patterns—specifically the “islands” of technical specialization versus the “bridges” of industrial application—provide the empirical basis for the necessity of a new evaluative structure.

The analysis presented in the results provides a detailed understanding of how HA research intersects with sustainability, well-being, and quality of life domains. We observed substantial variations in research volume, topical structure, and semantic linkages across these areas, highlighting both established and emerging pathways for integration. Building on these findings, we developed a nine-dimensional comparative performance framework through a two-stage analytical process. In the first stage, semantic clustering of the HA corpus revealed recurring performance descriptors—terms referring to capability enhancement, inclusion, resilience, and sustainability—across multiple research clusters. In the second stage, these descriptors were grouped into nine Key Performance Dimensions (KPDs) through an iterative content synthesis involving conceptual alignment with existing innovation and well-being frameworks (e.g., capability approach, sustainable innovation metrics). This mixed data-driven and conceptual approach ensured that the framework reflects both the empirical structure of current HA research and theoretical constructs of human-centric value creation.

The construction of this framework was further guided by the three most influential HA subclusters identified in the results—Industrial Worker Augmentation Systems (HA-5–1), Human-Building Interface Assessment Technologies (HA-10), and Assistive Augmentation Technologies (HA-27)—which served as empirical anchors linking the bibliometric findings to the evaluative dimensions. Each subcluster represents a distinct orientation within the HA landscape. Industrial Worker Augmentation Systems primarily informed the development of the Performance-Enhancing and Resilience-Enhancing categories, corresponding to KPDs on productivity, firm performance, and adaptability in harsh environments. Human-Building Interface Assessment Technologies aligned with Adaptive and Inclusive/Sustainable augmentation types, emphasizing environmental interaction, social inclusion, and ecological impact. Assistive Augmentation Technologies informed the Burden-Alleviating and Inclusive categories, reflecting KPDs associated with rehabilitation, pain reduction, and empowerment of individuals with disabilities. Together, these mappings ensure that the nine-dimensional framework is not abstract but empirically grounded in the structural and semantic patterns revealed by the citation and keyword network analyses.

Collectively, these empirical and conceptual foundations provide a coherent structure for assessing HA technologies across individual, organizational, and societal levels, thereby setting the stage for a detailed presentation of the comparative advantages framework across the nine KPDs.

### 5.1. Comparative advantages framework across KPDs

Drawing on the five clusters and nine identified performance dimensions represented in Fig 4, we categorize HA technologies into five main groups as shown in Table 3: Adaptive and Capability-Building Augmentation Technologies (enabling work in harsh environments and accelerated therapy, learning, or training), Burden-Alleviating Augmentation Technologies, Inclusive and Sustainable Augmentation Technologies (supporting social inclusion and decreasing environmental impact), Performance-Enhancing Augmentation Technologies (improving productivity and firm performance), and Resilience-Enhancing Augmentation Technologies (enhancing existing human abilities or creating entirely new abilities). Given that the impacts of these technologies span individual, organizational, and societal levels, it is practical and insightful to group these nine performance dimensions accordingly. Specifically, the individual level includes enabling work in harsh environments, enhancing human abilities, providing new abilities, supporting accelerated therapy or training, reducing pain and burden, and promoting social inclusion. At the organizational level, the focus is on improving productivity and firm performance. At the societal level, the emphasis is on decreasing environmental impact. Adopting this multi-level categorization aligns closely with previous research advocating for the necessity of assessing innovation and well-being outcomes across individual, organizational, and broader societal contexts to effectively capture comprehensive impacts and guide holistic innovation strategies [43]. To support side-by-side comparison, Table 3 organizes the nine KPDs under the five HA groups introduced above.

**Table 3:** Comparative framework for assessing HA technologies across nine KPDs.

Category	KPD	Tradeoffs of HA	Key Application Examples	Most Relevant cluster or subcluster
Individual Level				
Adaptive and Capability-Building Augmentation Technologies	1. Enable working in harsh environments	Combine enhanced safety and efficiency in harsh or extreme environments with human adaptability.	Life-support suits and astronauts augmentation [44] Underwater Superlimb for diving assistance [45]. Teleoperation system for contact-rich tasks such as removing deposited iron lumps in steel mills [46]. In-space additive manufacturing [47] and metal welding [48].	Supernumerary Robotic Limbs (HA-8–2) Control Systems for Human-Robot Physical Augmentation (HA-9)
	2. Assisting in accelerated therapy/learning/training	HA technologies expedite learning and rehabilitation by offering immersive and personalized experiences; however, they may require significant technological investment and user adaptation [49].	Performance optimization system for athletes in sports [14]. Virtual reality platforms for immersive educational experiences [50] Avatar personalization system for emotional influence in VR [51]. Skill acquisition tools for enhanced learning (e.g., piano) [16].	Emerging Technologies for Human Performance Enhancement and Virtual Augmentation (HA-3) Wearable Assistive Exoskeletons (HA-2–5)
Resilience-Enhancing Augmentation Technologies	3. Enhance human abilities	Augmented cognition and sensory enhancements improve decision-making speed and situational awareness, particularly in high-risk scenarios, yet ethical concerns regarding reliance on AI-driven insights and potential cognitive overload [4,52].	Cycling simulator for cognitive enhancement technologies in dangerous traffic situations [53]. AI-Interactive system for small arms weapon [52] Haptic eyes for sensory feedback and cognitive object recognition [4].	Brain-Computer Interfaces (HA-7–3) Emerging Technologies for Human Performance Enhancement and Virtual Augmentation (HA-3) Assistive Augmentation Technologies (HA-27)
	4.Giving humans new abilities	Augmented reality and supernumerary limb systems provide users with enhanced interaction capabilities beyond natural human limits, but they might require extensive training to develop proficiency [54]	AR-assisted surgical systems [55] AR and VR tools for enhanced telekinetic interaction and reality blending [56]	Digital Human-Machine Integration in Smart Manufacturing Systems (HA-5–2) Emerging Technologies for Human Performance Enhancement and Virtual Augmentation (HA-3)
Burden-Alleviating Augmentation Technologies	5. Reduce pain/burden	Alleviate physical strain and prevent injuries, particularly in labor-intensive industries and pain relief.	Sensor-based system for prevention, prediction, and personalized treatment of low back pain [57] A human-assistive robot for handling large payloads [58]	Exoskeleton Design and Control Systems (HA-1–5) Control Systems for Human-Robot Physical Augmentation (HA-9) Occupational Exoskeletons for Worker Assistance and Injury Prevention (HA-2–2) Hip Exoskeleton Control Systems (HA-1–1) Industrial Worker Augmentation Systems (HA-5–1)
Inclusive and Sustainable Augmentation Technologies	6. Support social inclusion	Assistive technologies enhance mobility and social participation for individuals with disabilities such as elderly people and people with diseases (e.g., ASD disorder, Parkinson)	Mobility assistance devices for individuals with walking disabilities [59] Assistive home avatars for user-friendly interaction in elderly care [60] Smart bracelet for enhancing human-human physical touch [61]	Ankle Exoskeleton Research (HA-1–2) Assistive Augmentation Technologies (HA-27) Robotic Assistive Technologies for Human Limb Augmentation and Rehabilitation (HA-2–1) Lower-limb Exoskeleton Systems (HA-1–6) Human-Building Interface Assessment Technologies (HA-10)
Organizational level				
Performance-Enhancing Augmentation Technologies	7. Increase productivity	Improve productivity by enhancing scalability and optimizing workflow.	Cognitive intelligence systems for industrial robots and smart manufacturing [62]. Wearable exoskeletons for workers in construction environments [63] Wearable structures that increase strength and endurance [64]	Soft Exosuit Technology (HA-1–4) Industrial Worker Augmentation Systems (HA-5–1)
	8. Increase firm performance	Enhance operational efficiency, and workforce accessibility.	Telepresence tools for remote collaboration [65]. AR technology for industrial services [66]	Emerging Technologies for Human Performance Enhancement and Virtual Augmentation (HA-3) Industrial Worker Augmentation Systems (HA-5–1)
Societal Level				
Inclusive and Sustainable Augmentation Technologies	9. Decrease environmental impact	Wearable computing and bio-inspired sensing technologies contribute to sustainability by integrating energy-efficient solutions, yet their production and disposal may introduce new ecological challenges	Solar shirt for noise pollution detection and energy-neutral wearable computing [67] Triboelectric nanogenerator wearables for sustainable IoT and green earth integration [68]	Human-Computer Interface Technologies for Social Interaction Enhancement (HA-16–2) Advanced Biomimetic Sensing Technologies (HA-15–2) Human-Building Interface Assessment Technologies (HA-10)

At the individual level, HA technologies primarily extend capabilities, enhance health, and improve well-being and quality of life. The first key performance dimension (1) emphasizes enabling humans to work safely in harsh or high-risk environments. Examples include life-support suits for astronauts [44] and underwater robotic limbs for divers [45]. These applications, found in clusters such as Supernumerary Robotic Limbs (HA-8–2) and Control Systems for Human-Robot Physical Augmentation (HA-9), provide adaptability and resilience beyond manual methods without sacrificing human control and flexibility [69]. A second dimension (2) advances accelerated therapy, learning, and training, with examples including athletic performance enhancement systems [14], immersive VR educational platforms [50], and personalized avatar systems for emotional engagement [51]. Clusters like Wearable Assistive Exoskeletons (HA-2–5) underline the personalized nature of these interventions, although user adaptation and technological investment remain challenges [49]. Cognitive enhancement (3) represents another critical strand, encompassing immersive traffic-safety simulations [53], AI-assisted interfaces [52], and haptic vision systems [4]. Despite their benefits, these raise ethical concerns around cognitive overload and reliance on automation [4,52]. Technologies from the Brain-Computer Interfaces (HA-7–3) cluster exemplify this intersection of cognitive performance and human adaptability.

Dimension 4 provides entirely new abilities beyond natural human limits, such as augmented reality (AR)-assisted surgery [55] and virtual reality (VR)-enabled telekinesis [56]. These innovations, detailed in Digital Human-Machine Integration in Smart Manufacturing Systems (HA-5–2) and Emerging Technologies for Human Performance Enhancement and Virtual Augmentation (HA-3), require users to develop proficiency with novel interfaces, thus representing a profound extension of human capability through technology [54]. Reducing physical strain (5) is equally central. Wearable exoskeletons for construction workers [63], sensor-based personalized low-back pain management systems [57], and payload-handling assistive robots [58] exemplify this dimension. Subclusters Exoskeleton Design and Control Systems (HA-1–5) and Control Systems for Human-Robot Physical Augmentation (HA-9) specifically address optimizing physical human-machine interactions, thus reducing injury risks and enhancing physical resilience. Finally, social inclusion (6) underscores HA’s humanistic purpose, empowering people with disabilities or age-related limitations [59–61] through adaptive assistive systems such as ankle exoskeletons and home-based augmentation platforms.

From an operational perspective, dimensions 7 and 8 illustrate how HA technologies enhance productivity and firm performance. Dimension 7 emphasizes productivity improvements, highlighting solutions like soft exosuit technology (Soft Exosuit Technology, HA-1–4) that augment physical output without replacing human judgment [62]. Unlike fully autonomous robots, which excel at repetitive tasks but face challenges adapting to dynamic conditions [70,71], HA solutions balance physical amplification with human cognitive flexibility. Dimension 8 addresses broader economic benefits, notably through telepresence technologies that enable remote collaboration (cluster 3: Emerging Technologies for Human Performance Enhancement and Virtual Augmentation) [65]. Such tools harness human creativity and expertise, overcoming geographical constraints and delivering greater scalability and adaptability than exclusively human teams or fully automated robotic systems.

From a societal standpoint, the ninth dimension captures environmental and sustainability impacts. Examples include solar-powered wearable sensors for noise pollution detection [67] and sustainable triboelectric nanogenerators for green IoT applications [68]. Subclusters like Human-Computer Interface Technologies for Social Interaction Enhancement (HA-16–2) and Advanced Biomimetic Sensing Technologies (HA-15–2) highlight HA’s unique ability to balance precision and efficiency, thus optimizing energy use and reducing environmental footprints. HA technologies, by integrating targeted sensor data and adaptive controls, present sustainable solutions superior to traditional low-tech or large-scale robotic methods. These nine dimensions motivate cross-domain combinations, which we examine next through technological convergence.

To facilitate the practical application of the nine-dimensional framework, we propose concrete, measurable indicators for each KPD in the Supplementary Material S6 Table in S1 File. These metrics enable systematic, quantitative assessment of HA technologies’ comparative advantages and societal impacts. The indicators draw from established methodologies in ergonomics, human factors engineering, organizational performance management, and sustainability assessment, ensuring that evaluations are grounded in validated measurement approaches.

### 5.2. Interdisciplinary synergies and technological convergence

Our comparative advantages framework emphasizes distinct performance dimensions across individual, organizational, and societal levels. However, our analysis also reveals significant opportunities for interdisciplinary synergies through technological convergence. At the individual level, assistive technologies for social inclusion (dimension 6) could incorporate cognitive monitoring systems (dimension 3) to develop adaptive interfaces for neurodiverse individuals. Subclusters such as Brain-Computer Interfaces (HA-7–3) and Assistive Augmentation Technologies (HA-27) illustrate this potential, aligning with existing research on human-robot symbiosis [20] and IoT-driven social sustainability [31]. Despite clear theoretical potential, these combinations remain underexplored, warranting further interdisciplinary investigation.

At the organizational level, convergence can overcome technical and operational limitations. For instance, combining VR-based training tools (dimension 2) with telepresence systems (dimension 8) may significantly reduce user adaptation costs [49] while enhancing productivity and remote collaboration capabilities. Such combinations could allow organizations to effectively scale HA technologies, facilitating smoother integration into existing workflows and broader adoption across various sectors.

Finally, at the societal level, integrating sustainability-oriented wearables (dimension 9) with biomimetic sensing technologies (HA-15–2) from industrial contexts (dimension 1) could substantially enhance energy efficiency. This convergence exemplifies the “human-tech symbiosis” paradigm [19], suggesting an optimal balance between advanced sensing capabilities and environmentally responsible practices. Realizing these interdisciplinary synergies requires coordinated innovation across academia, industry, and policy, particularly at the meso-level, where individual and systemic factors intersect to determine societal impacts. Realizing these synergies responsibly requires value-aligned design and governance, which we operationalize next through ethical integration within the framework.

### 5.3. Ethical integration and responsible innovation

As human augmentation (HA) technologies continue to permeate healthcare, industry, and daily life, ethical reflection must move from a peripheral consideration to an integral dimension of design and evaluation. Rather than treating ethics as an afterthought, it should be embedded within the comparative assessment of HA technologies—informing decisions about usability, autonomy, inclusiveness, and long-term societal value across all nine key performance dimensions (KPDs). Integrating ethical reasoning into the framework enables a more balanced evaluation of trade-offs between performance gains and human welfare, ensuring that technological advancement remains aligned with human-centric principles. For instance, KPDs 3 (enhancing human abilities), 6 (supporting social inclusion), and 9 (decreasing environmental impact) are particularly sensitive to ethical trade-offs, underscoring the need for explicit evaluation criteria embedded in these dimensions.

Operationalizing ethical safeguards within HA systems can be achieved through real-time monitoring and adaptive feedback mechanisms. For example, EEG-based emotion and stress detection [72] can serve as an ethical control layer by identifying cognitive overload and maintaining user well-being during augmented operation. Embedding such monitoring capabilities directly into device architecture exemplifies how ethics can be translated into actionable design criteria. These approaches are particularly crucial in high-stakes contexts—such as medical diagnostics, defense systems, and critical infrastructure—where excessive automation or overreliance on AI-driven decision interfaces can compromise moral accountability and situational awareness [52].

Beyond technical safeguards, ethical integration also requires institutional and procedural mechanisms. Incorporating responsible research and innovation (RRI) principles—such as reflexivity, transparency, and stakeholder participation—can align technological development with societal expectations. Embedding these principles into HA evaluation processes encourages multidisciplinary oversight that bridges engineers, ethicists, policymakers, and end users. By systematically mapping each HA technology’s ethical footprint across individual, organizational, and societal levels, the framework transforms ethics from a reactive safeguard into a proactive tool for value alignment.

Ultimately, this integration supports a dynamic model of *responsible innovation*—one capable of identifying early ethical warning signals, guiding equitable deployment, and fostering public trust. When combined with the framework’s performance dimensions, ethical integration provides a normative compass for navigating the accelerating evolution of HA technologies, ensuring that progress enhances rather than undermines human flourishing. Building on this ethical foundation, the following subsections identify emerging frontiers where HA development can advance in alignment with these normative principles.

### 5.4. Emerging frontiers and future research priorities

#### 5.4.1. Individual-level opportunities: Behavioral and recreational applications.

Our analysis identifies two underexamined yet promising domains at the individual level: systematic human behavioral change and leisure or recreational applications. While current HA research predominantly emphasizes clinical or industrial applications, preliminary studies indicate broader implications for individual behavior and well-being. For example, Extended Reality (XR) systems designed for cognitive load monitoring [73] and AI-driven decision tools [74] demonstrate HA’s latent ability to influence behavior positively, such as improving workplace safety or promoting sustainable habits. Other examples include heatmaps and pointing tools in collaborative AI systems, as well as HA technologies designed to help people take medications regularly [75]. These technologies guide, and sometimes even change, human decisions, showing that augmented interfaces can strongly influence user behavior [74]. Further, VR collaboration frameworks [76] and emotionally intelligent interfaces [77] highlight how augmentation technologies might reshape individual decision-making processes and enhance social interactions.

Parallel opportunities exist within leisure and recreation, transcending purely utilitarian applications. Technologies originally developed for industrial scenarios, such as cycling simulators [53] and AI-enhanced sports interfaces [52], could be effectively repurposed for immersive fitness and adaptive gaming experiences. Although existing studies have hinted at HA potential within mobile gaming [78] and adaptive sports [79], explicit research on systematic leisure and recreational applications remains sparse. Therefore, future research should deliberately explore HA’s long-term influence on behavioral modification and enrichment of leisure activities, explicitly framing these interventions as distinct research domains.

#### 5.4.2. Organizational-level opportunities: technical scalability and inclusive design.

At the organizational level, strategic priorities include overcoming technical scalability barriers and fostering inclusive design practices. Energy-efficient solutions, such as triboelectric nanogenerators [67], can lower operational costs and environmental footprints, enhancing overall organizational sustainability. Furthermore, modular design architectures that allow incremental upgrades could mitigate risks related to technological obsolescence, providing long-term organizational value.

Inclusive design of HA solutions represents a critical area for organizational commercialization and adoption. HA systems like VR esports platforms and assistive exoskeletons (HA-1–2: Ankle Exoskeleton Research) for adaptive sports demonstrate considerable potential. These technologies could democratize augmented leisure and professional opportunities, promoting equity and accessibility. Realizing this potential, however, demands explicit interdisciplinary cooperation to accommodate diverse physical, cognitive, and socioeconomic user requirements.

#### 5.4.3. Societal-level opportunities: Sustainability and governance.

At the societal level, HA development must prioritize sustainability governance. Lifecycle assessments of HA products, ranging from solar-powered wearables to broader IoT ecosystems, are essential for establishing evidence-based eco-design standards. Such standards ensure that environmental impacts remain integral to the innovation process, aligning HA advances with global sustainability and equity imperatives.

Moreover, equitable access frameworks should be developed to ensure that assistive technologies reach low-resource and marginalized settings [58]. Achieving this requires robust policy-academia-industry alignment, exemplified by initiatives combining VR training tools with telepresence for critical applications such as disaster response [50,65]. Policymakers should adopt participatory methods, such as literature-based discovery (LBD) frameworks [33], to ensure HA development aligns closely with the UN Sustainable Development Goals, especially in sensitive domains like healthcare and defense.

### 5.5. Macroeconomic impact of HA technology adoption

A critical yet largely unexplored research direction involves assessing the macroeconomic impacts of widespread HA technology adoption. Key research questions include identifying which occupations are most susceptible to HA interventions and which economic sectors will experience the most significant productivity shifts. Such insights are crucial to understand how augmentation-driven efficiency gains might influence employment dynamics, wage distribution, and inter-sectoral dependencies.

Future empirical work could apply multi-sectoral and general equilibrium modeling frameworks to simulate these dynamics at national and international scales. For example, Computable General Equilibrium (CGE) models can capture feedback loops between technology adoption, factor substitution, and consumption patterns, revealing potential outcomes such as productivity-induced growth or displacement effects. Alternatively, Input–Output (IO) and Social Accounting Matrix (SAM) approaches can be employed to estimate sectoral spillover effects, tracing how HA-induced productivity changes in one industry propagate through intermediate demand chains to influence output, income, and employment across others. These models can also quantify the indirect environmental and energy implications of HA diffusion, thus integrating economic and sustainability outcomes.

Hypothetical scenarios further illustrate this potential. For instance, large-scale adoption of assistive exoskeletons in construction and manufacturing could raise labor productivity while reducing occupational injury costs. This, in turn, could alter capital–labor ratios, decrease insurance expenditures, and increase investment in complementary digital systems, ultimately boosting output in upstream sectors such as robotics, materials science, and data analytics. Similarly, cognitive augmentation tools in service industries—such as finance, education, and healthcare—could lead to measurable improvements in decision efficiency, with cascading effects on GDP growth, service quality, and innovation diffusion.

Beyond quantifying these direct and indirect effects, macroeconomic modeling can inform policy design by identifying trade-offs between technological substitution and social inclusion. Scenario-based analyses can, for example, evaluate how different policy instruments—such as tax incentives, R&D subsidies, or retraining programs—affect the overall welfare gains from HA adoption. Incorporating distributional impact modules within such models would further allow policymakers to anticipate inequalities in access, skill polarization, or regional disparities that may arise from uneven technological diffusion.

By embedding HA within a systems-level analytical framework, future research can bridge the current gap between technological innovation and economic policy, providing a foundation for evidence-based industrial, labor, and social strategies that maximize the societal return on human augmentation technologies. We now translate these system-level insights into near-term implications and constraints for deployment.

### 5.6. Practical implications and study limitations

Real-world deployments of human augmentation (HA) technologies validate our nine-dimensional framework by demonstrating benefits across individual, organizational, and societal levels. Academic research further supports these impacts.

**Industrial exoskeletons**: Ford Motor Company’s use of EksoVest exoskeletons reduced worker discomfort and fatigue, while the ZeroG tool-holder improved drilling productivity by 200–300%. Ford’s broader ergonomic program, integrating exoskeletons and virtual manufacturing tools, contributed to an 83% decline in lost-time injuries since 2005 [80]. These results align with framework dimensions on physical burden reduction (5), productivity gains (7), and societal cost savings (9). Academic reviews by Fournier et al. [81] and De Looze et al. [82] confirm exoskeletons’ effectiveness in reducing physical loads and preventing musculoskeletal disorders, while McFarland and Fischer [83] highlight their role in enhancing productivity.

**Augmented reality (AR) training**: A pharmaceutical firm employed PTC’s Vuforia Expert Capture to digitize procedures, cutting training time by over 50% and projecting US$27 million annual savings per factory [84]. This reflects assisting in accelerated learning (2), organizational productivity (7), and broader environmental/economic benefits (9). Studies by Martins et al. [85] and Gualtieri et al. [86] show AR’s ability to significantly reduce learning time and enhance knowledge retention, with Webel et al. (2013) [87] noting its contribution to time and cost savings.

**Virtual reality (VR) immersive learning**: Walmart’s VR training increased employee performance by 10% and generated annual savings exceeding US$400 million; Honeywell achieved a 40% reduction in training time using similar methods. Unilever’s VR-based leadership program reported a 30% boost in retention [88]. These outcomes demonstrate assisting in accelerated learning (2), enhancing human abilities (3), and multi-level productivity and cost benefits (7, 9). Holusa et al. [89] and Radhakrishnan and Koumaditis [90] corroborate VR’s efficacy in reducing training time and improving performance, while Koumaditis et al. [91] underscores its cost-effectiveness.

Collectively, these cases confirm that HA technologies deliver the multi-level advantages predicted by our framework, from reducing physical strain to enhancing skills and generating significant economic value. Mapping such evidence to our dimensions helps organizations anticipate impacts and guide responsible adoption. The development and validation of multi-dimensional frameworks, as discussed by Heard and Adams [92] and Alfano et al. [93], further solidify the academic rigor behind such approaches.

This study acknowledges several methodological limitations that may affect the generalizability and comprehensiveness of its findings. The exclusive reliance on academic literature may underrepresent significant developments in HA technologies emerging from industry and commercial sectors. Consequently, our framework may better characterize research-stage and clinically-oriented HA technologies than commercially mature products. The prominence of Industrial Worker Augmentation Systems (HA-5–1) in our findings likely reflects substantial academic-industry collaboration in this domain, whereas consumer-oriented HA applications (e.g., fitness wearables, gaming peripherals) may be systematically underrepresented. Future research should triangulate academic findings with patent databases (e.g., USPTO, EPO, WIPO), industry market reports, and regulatory approval documents (e.g., FDA 510(k) clearances for medical devices) to capture the full innovation landscape. Such multi-source approaches could reveal implementation gaps between academic research and industry practice, enhance the framework’s commercial applicability, and provide real-world validation of the theoretical linkages identified in this study.

Finally, the semantic similarity analysis, while methodologically robust, is inherently limited by its reliance on textual content. It may overlook factors such as real-world implementation challenges, user acceptance, and performance metrics critical to successful adoption. While this study does not directly assess these aspects, it compensates by highlighting the need for future empirical work grounded in lived user experience, field trials, and human-centered design evaluations.

### 5.7. Future work and practical application of the framework

While this study established a data-driven and conceptually grounded framework, empirical validation remains essential to refine its applicability. Future research should operationalize the nine performance dimensions through case studies, longitudinal user testing, and human-centered design evaluations across diverse HA contexts such as rehabilitation, industrial safety, and digital learning. Such efforts can confirm the internal consistency of the framework and quantify trade-offs between individual, organizational, and societal benefits.

In practice, the framework can serve as a decision-support tool for innovators, policymakers, and investors seeking to prioritize HA technologies that maximize positive societal impact. For example, technology developers may use it to benchmark prototypes across multiple impact levels, while policymakers can employ it to evaluate whether emerging HA initiatives align with national well-being or sustainability agendas. Integrating the framework into participatory innovation processes, such as co-design workshops or responsible research and innovation (RRI) assessments, would provide iterative feedback loops that link conceptual insights with real-world performance.

Beyond methodological validation, the framework can also serve as a foresight and anticipatory governance tool to explore alternative development trajectories of HA. Future studies could simulate both optimistic and pessimistic scenarios; for instance, comparing pathways where augmentation promotes equity, accessibility, and sustainability versus those where it amplifies inequality, cognitive dependency, or ecological burden. By mapping these trajectories across the nine performance dimensions, researchers can identify which KPDs act as early indicators of imbalance or societal risk. This scenario-based application positions the framework as a strategic instrument for guiding HA development under uncertainty, enabling decision-makers to anticipate potential trade-offs and steer innovation toward trajectories that reinforce well-being, resilience, and sustainable growth while mitigating unintended consequences. These directions reinforce the broader contribution of this study in establishing a data-driven, ethically anchored, and policy-relevant framework for guiding the evolution of human augmentation research and practice.

## 6. Conclusion

This study presented a comprehensive, multilevel assessment of how Human Augmentation (HA) technologies intersect with societal priorities, including sustainability, well-being, and quality of life. Through citation network analysis and semantic similarity mapping, we identified that while HA research remains distinct from these social domains, it possesses deep technical specialization and three pivotal areas of convergence: Industrial Worker Augmentation, Human-Building Interfaces, and Assistive Technologies. Based on these empirical insights, we developed a novel nine-dimensional performance framework that categorized HA technologies into five functional groups across individual, organizational, and societal levels. By validating this framework against real-world applications, this research established a scalable tool for prioritizing innovations, evaluating trade-offs, and aligning technological adoption with critical global goals such as equity, resilience, and environmental sustainability.

Based on these findings, we propose clear and actionable recommendations for future research and practice. First, researchers should expand beyond academic literature by integrating patent data and industry reports to capture commercial advancements and bridge the gap between theoretical models and market realities. Second, the proposed framework requires empirical operationalization through longitudinal user trials and case studies—particularly in rehabilitation and industrial safety—to quantify the real-world trade-offs between productivity and human well-being. Third, future scholarship must investigate the macroeconomic implications of HA adoption, utilizing general equilibrium models to forecast labor displacement and cross-sectoral productivity shifts. Finally, for practitioners and policymakers, we recommend utilizing the framework as a decision-support tool to benchmark prototypes and guide anticipatory governance. Integrating these performance dimensions into participatory processes, such as co-design workshops, will ensure that emerging HA initiatives align with national sustainability agendas and address ethical risks early in the innovation cycle.

Despite these contributions, several limitations warrant acknowledgment. This study relied exclusively on academic literature, which may underrepresent private-sector advancements and non-English scholarship. Furthermore, the semantic similarity analysis depends on textual content, potentially overlooking real-world implementation challenges such as user acceptance and physical performance metrics. These constraints highlight the necessity of the multi-source, empirical approaches recommended above to fully capture the evolving landscape of human augmentation.

Strategically guided and ethically responsible HA could evolve from a niche field into a transformative driver of inclusive progress. To realize this potential, sustained collaboration among academia, industry, and government will be essential. Such efforts must ensure HA technologies enhance human capabilities, uphold ethical principles, and address urgent global challenges.

## Supporting information

S1 File Dataset formation, analysis parameters, and candidate indicators for KPDs. Also contain S1-S6 Table and S1 Figure.
(DOCX)
