# Supplementary material for: Mapping human augmentation technologies for societal impact: A multilevel framework for classification and innovation potential
Source: PLoS One. 2026 Feb 25;21(2):e0343292. doi: 10.1371/journal.pone.0343292 (PMC12935241; doi:10.1371/journal.pone.0343292)
Supplement: S1 File — Also contain S1-S6 Table and S1 Figure. (DOCX) [file pone.0343292.s001.docx]

Supplementary Information

***Mapping human augmentation technologies for societal impact: A multilevel framework for classification and innovation potential***

# 1. Dataset formation

Table S1 documents the progression from initial Web of Science query results to final analyzed networks. "Incomplete/Duplicated" include articles with insufficient bibliographic information for citation matching or identified duplicates (missing DOI, author names, publication year, journal title, volume, or issue number). " Not in largest component" represents articles not connected to the main citation network. Final node counts correspond to all records used for clustering and semantic similarity assessments.

Table S1. Dataset construction and filtering process for citation network analysis.

| **Dataset** | **Query** | **Hits** | **Incomplete/ Duplicated** | **Not in largest component** | **Nodes** |
| --- | --- | --- | --- | --- | --- |
| HA | TS= (“human* augmentation" OR "human* enhancement" OR "augmenting human*" OR "enhancing human*”) AND (DT="article") AND (WC=see list below) + Citing Articles | 10,957 | -346 | -4,327 | 6,284 |
| Sustainability | (TI="sustainability") AND (PY=(2015-2025)) AND (DT="article") | 41,400 | -489 | -12,783 | 28,128 |
| Wellbeing | (TI = ("wellbeing" OR "well-being" OR "well being")) AND (PY=(2015-2025)) AND (DT="article") | 35,111 | -381 | -8,449 | 26,281 |
| Quality of Life | (TI = "quality of life") AND (PY=(2015-2025)) AND (DT="article") | 54,077 | -35 | -10,267 | 43,775 |

Note: Data was retrieved on December 12, 2024. The years queried include 2025 to ensure that records with future publication dates, which are classified as early-access within the database, are also incorporated.

The Human Augmentation dataset was filtered using the Web of Science Categories (WC) field to ensure relevance to engineering, computer science, physical sciences, and related interdisciplinary domains. The following categories (Table S2) were included in the search filter. For the three social domain datasets (Sustainability, Wellbeing, and Quality of Life), no category filtering was applied given their inherently multidisciplinary nature.

Table S2. Web of Science categories used for Human Augmentation dataset filtering.

| "Agricultural Engineering", | "Engineering, Civil", | "Mathematics, Interdisciplinary Applications", |
| --- | --- | --- |
| "Astronomy & Astrophysics", | "Engineering, Electrical & Electronic", | "Mathematics", |
| "Biochemistry & Molecular Biology", | "Engineering, Environmental", | "Mechanics", |
| "Biophysics", | "Engineering, Geological", | "Medical Laboratory Technology", |
| "Biotechnology & Applied Microbiology", | "Engineering, Industrial", | "Metallurgy & Metallurgical Engineering", |
| "Cell & Tissue Engineering", | "Engineering, Manufacturing", | "Nanoscience & Nanotechnology", |
| "Chemistry, Analytical", | "Engineering, Marine", | "Nuclear Science & Technology", |
| "Chemistry, Applied", | "Engineering, Mechanical", | "Physics, Applied", |
| "Chemistry, Inorganic & Nuclear", | "Engineering, Multidisciplinary", | "Physics, Atomic, Molecular & Chemical", |
| "Chemistry, Medicinal", | "Engineering, Ocean", | "Physics, Condensed Matter", |
| "Chemistry, Multidisciplinary", | "Engineering, Petroleum", | "Physics, Fluids & Plasmas", |
| "Chemistry, Organic", | "Food Science & Technology", | "Physics, Mathematical", |
| "Chemistry, Physical", | "Geochemistry & Geophysics", | "Physics, Multidisciplinary", |
| "Computer Science, Artificial Intelligence", | "Green & Sustainable Science & Technology", | "Physics, Nuclear", |
| "Computer Science, Cybernetics", | "Imaging Science & Photographic Technology", | "Physics, Particles & Fields", |
| "Computer Science, Hardware & Architecture", | "Materials Science, Biomaterials", | "Psychology, Mathematical", |
| "Computer Science, Information Systems", | "Materials Science, Ceramics", | "Quantum Science & Technology" |
| "Computer Science, Interdisciplinary Applications", | "Materials Science, Characterization & Testing", | "Robotics", |
| "Computer Science, Software Engineering", | "Materials Science, Coatings & Films", | "Social Sciences, Mathematical Methods", |
| "Computer Science, Theory & Methods", | "Materials Science, Composites", | "Telecommunications", |
| "Construction & Building Technology", | "Materials Science, Multidisciplinary", | "Thermodynamics", |
| "Electrochemistry", | "Materials Science, Paper & Wood", | "Transportation Science & Technology", |
| "Engineering, Aerospace", | "Materials Science, Textiles", |  |
| "Engineering, Biomedical", | "Mathematical & Computational Biology", |  |
| "Engineering, Chemical", | "Mathematics, Applied", |  |

Table S3. Data collection and export specifications for reproducibility.

| ***Parameter*** | ***Specification*** |
| --- | --- |
| Database | Web of Science Core Collection |
| Collection date | 12-Dec-24 |
| Export format | “Tab-delimited file” (plain text) |
| Export scope | “Full records and cited references” |
| Metadata fields used | UT (Unique identifier), PY (Publication year), Z9 (Times cited count), TI (Title), AB (Abstract), CR (Cited references), DI (Digital Object Identifier), SO (Source/journal), VL (Volume), IS (Issue), BP (Beginning page), AU (Authors), WC (Web of Science categories) |
| Document type filter | Article (applied to all four datasets) |
| Timespan filter | HA dataset: All years available; Social domain datasets: 2015-2025 (Past 10 years) |

Note on citation matching: Cited references (CR field) were matched to articles within each dataset using DOI when available. For articles without DOI, matching was performed by comparing AU (first author surname), PY, SO, VL, and IS fields, requiring exact matches on all five fields to confirm a citation link.

# 2. Robustness analysis

To ensure our clustering results are methodologically robust, we systematically compared (1) Louvain at different resolution parameters (γ = 0.1, 0.5, 1, 1.5, 2), (2) Walktrap (at different step lengths), (3) Infomap, and (4) Fast Greedy. Performance was assessed using modularity scores, which measure the quality of network partitioning by quantifying the density of connections within communities versus between communities. Higher modularity indicates stronger community structure. Results show that Louvain with γ = 1.0 achieves the highest. Table S4 below presents modularity values for each algorithm-parameter combination applied to the Human Augmentation dataset.

Table S4. Sensitivity analysis of clustering algorithms and parameters.

| ***Clustering Algorithm*** | ***Clusters*** | ***Modularity*** |
| --- | --- | --- |
| Louvain (resolution = 2) | 41 | 0.791278 |
| Louvain (resolution = 1.5) | 34 | 0.8140279 |
| Louvain (resolution = 1) | 30 | 0.9012189 |
| Louvain (resolution = 0.5) | 18 | 0.8482404 |
| Louvain (resolution = 0.1) | 6 | 0.9002799 |
| Walktrap (steps=3) | 155 | 0.7935646 |
| Walktrap (steps=4) | 128 | 0.8015609 |
| Walktrap (steps=5) | 110 | 0.8109015 |
| Infomap | 207 | 0.7857076 |
| Fast Greedy | 27 | 0.8368337 |

To assess the robustness of our semantic linkage analysis to text-embedding model selection, we computed semantic similarity matrices using five different pre-trained transformer models and examined their pairwise correlations. The models tested represent different architectures and training objectives:

- all-MiniLM-L6-v2 (our primary model): Compact sentence transformer optimized for semantic similarity tasks (384 dimensions)
- all-MiniLM-L12-v2: Deeper variant with 12 transformer layers (384 dimensions)
- gsarti/scibert-nli: A model trained on papers from the corpus of semanticscholar.org (768 dimensions)
- BAAI/bge-base-en-v1.5: Recent state-of-the-art general-purpose embedding model (768 dimensions)

For each model, we generated embeddings for all cluster texts (aggregated titles and abstracts) and computed cosine similarity matrices between HA subclusters and social domain subclusters. We then calculated Pearson correlation coefficients between the resulting similarity matrices.

Table S5. Correlation of semantic similarity matrices across embedding models.

| ***Model*** | ***all-MiniLM-L6-v2*** | ***all-MiniLM-L12-v2*** | ***gsarti/scibert-nli*** | ***BAAI/bge-base-en-v1.5*** |
| --- | --- | --- | --- | --- |
| ***all-MiniLM-L6-v2*** | 1 | - | - | - |
| ***all-MiniLM-L12-v2*** | 0.976 | 1 | - | - |
| ***gsarti/scibert-nli*** | 0.902 | 0.884 | 1 | - |
| ***BAAI/bge-base-en-v1.5*** | 0.879 | 0.856 | 0.888 | 1 |

# 3. Similarity threshold selection

Distribution of cosine similarity scores across all HA-social domain cluster pairs. We chose a similarity score >= 0.3609 for "strong" semantic linkage, representing the outlier pairs. This threshold ensures that highlighted connections represent the top of observed cross-domain relationships, capturing substantive thematic overlaps while excluding weaker or more generic associations. The top 50 connections visualized in Figure 3 of the main manuscript all exceed the 0.498 threshold, representing semantic linkages in the 94th percentile or higher of all possible HA-social domain relationships.


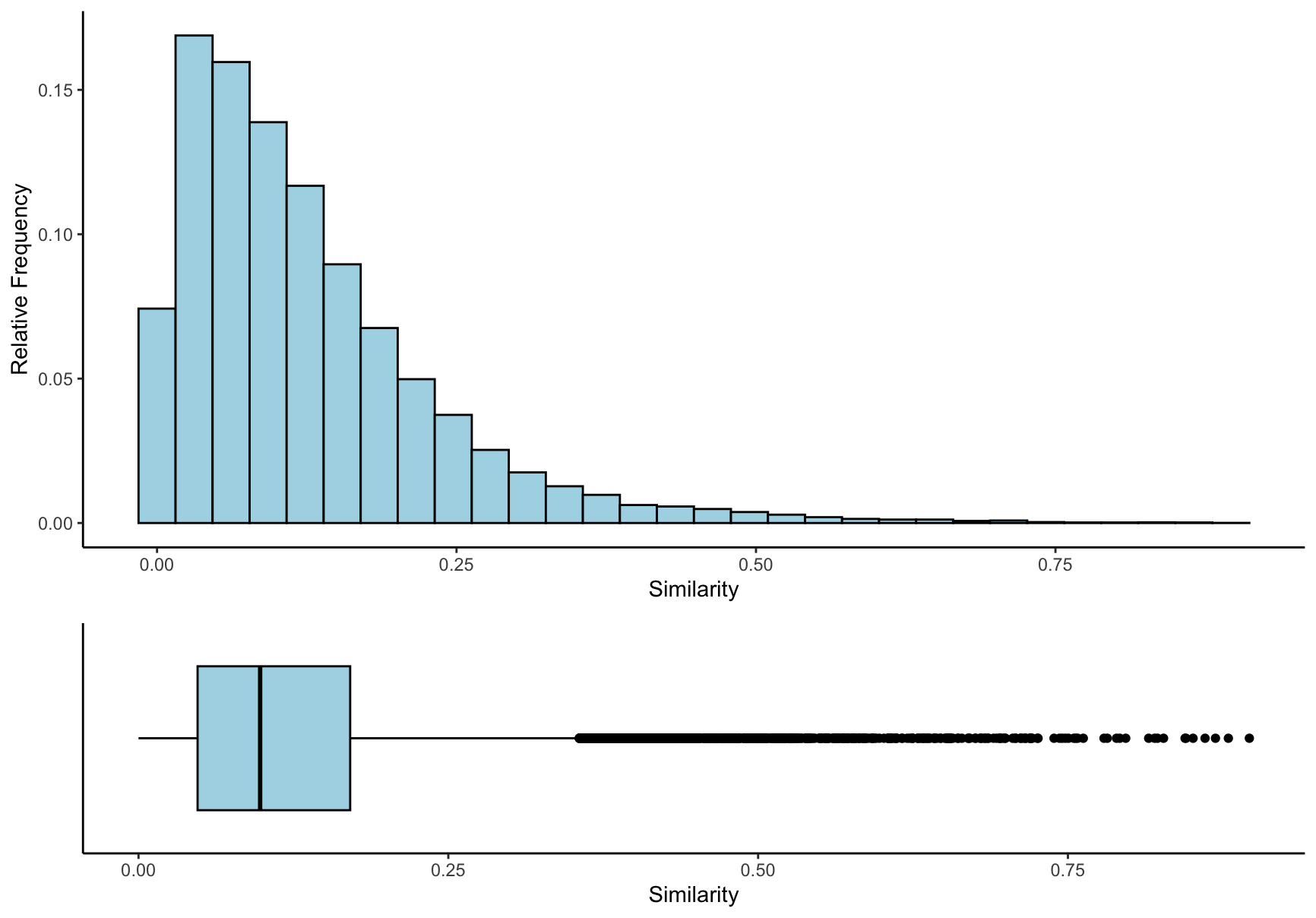


Figure S1. Distribution of semantic similarity scores between HA and social domain subclusters. **Upper panel (Histogram):** Relative frequency distribution of cosine similarity scores computed between all 60 HA subclusters and all subclusters from the three social domains (Sustainability: 257 subclusters; Wellbeing: 186 subclusters; Quality of Life: 296 subclusters), totaling 44,340 pairwise comparisons. The distribution is right-skewed, indicating that most cross-domain cluster pairs have low to moderate semantic similarity. **Lower panel (Boxplot):** Summary statistics of the similarity distribution. The lowest similarity is 1.285225e-07, the median similarity is 1.024717e-01, and the maximum similarity is 0.9132788, with the interquartile range spanning 5.077780e-02 (Q1) to 1.748662e-01 (Q3).

# 4. Candidate indicators for each KPD of the nine-dimensional framework

The following presents candidate indicators to the KPD presented in Table 3 in the main manuscript. Indicator selection should be tailored to specific HA application contexts and stakeholder priorities. Multi-KPD assessment is recommended to capture trade-offs (e.g., productivity gains vs. environmental costs) and synergies (e.g., pain reduction improving both individual well-being and organizational productivity).

Table S6. Potential indicators, measurement methods, and examples for the KPD

| **Candidate Measurable Indicators** | **Measurement Methods / Data Sources** | **Example Applications** |
| --- | --- | --- |
| **Individual -** 1. Enable working in harsh environments | | |
| • Incident/injury rates in hazardous zones  • Time safely spent in extreme conditions  • Physiological stress indicators (heart rate variability, cortisol)  • Task completion success rate | • Occupational safety databases (OSHA, HSE)  • Wearable biosensor data  • Environmental monitoring systems  • Task performance logs | Astronaut augmentation systems, underwater operations, nuclear/chemical facility work |
| **Individual -** 2. Assisting in accelerated therapy/learning/training | | |
| • Training time reduction (%)  • Time to competency (hours/days)  • Skill retention rate at follow-up  • Error rate reduction  • Cost per trained individual | • Pre-post training assessments  • Learning management systems  • Standardized skill evaluation protocols  • Training cost accounting | VR surgical training, AR industrial maintenance training, rehabilitation therapy protocols |
| **Individual -** 3. Enhance human abilities | | |
| • Reaction time improvement (ms)  • Decision accuracy increase (%)  • Situational awareness scores (SAGAT, SART)  • Cognitive load reduction (NASA-TLX)  • Detection threshold enhancement | • Standardized cognitive tests  • Eye-tracking and attention metrics  • Neurophysiological measures (EEG, fNIRS)  • Performance benchmarking | Cognitive enhancement for high-stakes decision-making, sensory augmentation for inspection tasks |
| **Individual -** 4. Giving humans new abilities | | |
| • Number of novel tasks enabled  • Success rate in previously impossible tasks  • Precision/accuracy in extended operations  • Range of motion/capability expansion | • Capability assessment protocols  • Task analysis and time studies  • Clinical functional assessments  • User studies and field trials | AR-assisted surgery enabling new procedures, supernumerary limbs for complex manipulation |
| **Individual -** 5. Reduce pain/burden | | |
| • Pain intensity scores (VAS, NRS, McGill Pain Questionnaire)  • Musculoskeletal injury incidence rate  • Muscle fatigue (EMG amplitude, frequency)  • Joint loading reduction (%)  • Metabolic cost (oxygen consumption, heart rate)  • Workers' compensation claims | • Clinical pain assessment instruments  • Biomechanical analysis (motion capture, force plates)  • Electromyography (EMG)  • Occupational health records  • Insurance/HR databases | Industrial exoskeletons for manual handling, assistive devices for chronic pain management |
| **Individual -** 6. Support social inclusion | | |
| • Functional independence measures (FIM, Barthel Index)  • Activities of daily living (ADL) scores  • Quality of life scales (SF-36, EQ-5D, WHO-QOL)  • Social participation indices  • Employment rate for assisted populations  • Accessibility compliance scores | • Validated clinical assessment tools  • Rehabilitation outcome measures  • Quality of life surveys  • Employment statistics  • Accessibility audits | Mobility aids for elderly, communication devices for speech impairments, assistive home technologies |
| **Organizational -** 7. Increase productivity | | |
| • Output per worker-hour (units/hour)  • Task completion time reduction (%)  • Cycle time improvement  • Throughput increase  • Defect/error rate reduction  • Capacity utilization rate | • Production management systems  • Time and motion studies  • Manufacturing execution systems (MES)  • Quality control databases  • Process mining analytics | Exoskeletons in manufacturing, AR guidance systems for assembly, cognitive assistance for complex procedures |
| **Organizational -** 8. Increase firm performance | | |
| • Return on investment (ROI, %)  • Cost savings ($/year)  • Revenue per employee  • Labor productivity index  • Downtime reduction (%)  • Employee retention rate  • Training cost reduction | • Financial reporting systems  • Enterprise resource planning (ERP) data  • HR analytics platforms  • Balanced scorecard metrics  • Cost-benefit analysis | Telepresence systems for remote expertise, VR training reducing travel/facility costs, AR maintenance reducing downtime |
| **Societal -** 9. Decrease environmental impact | | |
| • Energy consumption per operation (kWh)  • Carbon footprint (kg CO₂-eq)  • Life cycle assessment (LCA) scores  • Material intensity (kg/unit function)  • Waste generation rate  • Energy efficiency ratio  • Circular economy indicators (recyclability, repairability) | • Energy monitoring systems  • LCA software (SimaPro, openLCA)  • Carbon accounting frameworks (GHG Protocol)  • Environmental product declarations (EPD)  • ISO 14040/14044 standards  • Sustainability reporting (GRI, SASB) | Energy-harvesting wearables, IoT sensors for resource optimization, building-interface systems for energy efficiency |

*Abbreviations: OSHA = Occupational Safety and Health Administration; HSE = Health, Safety, Environment;VAS = Visual Analog Scale; NRS = Numeric Rating Scale; SAGAT = Situation Awareness Global Assessment Technique; SART = Situation Awareness Rating Technique; NASA-TLX = NASA Task Load Index; EEG = Electroencephalography; fNIR = Functional near-infrared spectroscopy; EMG = Electromyography; FIM = Functional Independence Measure; SF-36 = 36-Item Short Form Health Survey; EQ-5D = EuroQol Five Dimensions; WHO-QOL = World Health Organization Quality of Life; ADL = Activities of Daily Living; LCA = Life Cycle Assessment; GHG = Greenhouse Gas; GRI = Global Reporting Initiative; SASB = Sustainability Accounting Standards Board.*
